# To Treat or Not to Treat: The Role of Adjuvant Radioiodine Therapy in Thyroid Cancer Patients

**DOI:** 10.1155/2012/707156

**Published:** 2012-11-01

**Authors:** Marilee Carballo, Roderick M. Quiros

**Affiliations:** ^1^Department of Surgery, St. Luke's University Health Network, 801 Ostrum Street, Bethlehem, PA 18015, USA; ^2^Division of Surgical Oncology, Cancer Care Associates, St. Luke's University Health Network, 1872 Riverside Circle, Easton, PA 18045, USA

## Abstract

Radioactive iodine (RAI) is used in treatment of patients with differentiated papillary and follicular thyroid cancer. It is typically used after thyroidectomy, both as a means of imaging to detect residual thyroid tissue or metastatic disease, as well as a means of treatment by ablation if such tissue is found. In this paper, we discuss the indications for and the mechanisms of RAI in the treatment of patients with thyroid cancer. We discuss the attendant risks and benefits that come with its use, as well as techniques used to optimize its effectiveness as an imaging tool and a therapeutic modality.

## 1. Introduction

Thyroid carcinoma is the most common endocrine malignancy in the United States, with an estimated incidence of 56,460 in 2012 [1]. Most cases are comprised of differentiated subtypes, specifically papillary and follicular thyroid cancer. Surgical resection by way of thyroidectomy is the mainstay of treatment for this disease. However, adjuvant therapy in the form of radioactive iodine (RAI) is often administered as a means of reducing the risk of tumor recurrence and to facilitate future cancer surveillance. In this paper, we review indications for radioiodine ablation (RAI) as an adjuvant treatment for patients with papillary and follicular thyroid cancer.

## 2. Clinical Scenario

We present the case of a 44-year-old patient who had an uncomplicated, self-resolving upper respiratory tract infection. After resolution of her upper respiratory tract infection, she continued to have anterior neck swelling which was thought to be persistent lymphadenopathy from the recent infection. Because the swelling persisted longer than expected after infection, a cervical ultrasound was done which showed a 2 cm solid nodule in her left thyroid lobe. The remainder of the ultrasound exam, including cervical nodes, was normal. She subsequently underwent an ultrasound-guided fine needle aspiration (FNA) of this nodule, which was confirmed as a follicular thyroid neoplasm. She therefore underwent an uneventful left hemithyroidectomy and isthmectomy. The final pathology showed a stage I papillary microcarcinoma (3 foci, with the largest focus measuring 3 mm in greatest extent), limited to the thyroid, with negative margins, and no involved lymph nodes. The original 2 cm target lesion proved to be a benign adenomatoid nodule. Another 7 weeks later, she returned for a flexible laryngoscopy to assess vocal cord function, which was normal on exam, and completion right thyroidectomy. In the absence of clinically evident nodes along with a negative interrogation on the initial ultrasound, a level VI node dissection was not done. The final pathology report was notable for a single 3.5 mm focus of papillary microcarcinoma, follicular variant, in the right lobe. After a discussion on the risks and benefits of adjuvant RAI ablation, she did not undergo ablative treatment, given the size and extent of her cancer. Postoperative calcium levels were normal upon her recovery from the completion thyroidectomy.

As part of routine surveillance, the patient underwent interval serum testing and cervical ultrasounds. Two years after her final surgery, the ultrasound revealed prominent right anterior jugular lymph nodes with an associated punctuate microcalcification, with the largest node measuring 2.5 × 0.6 × 1.0 cm. An FNA of this lymph node showed metastatic papillary cancer. These findings led to a right modified radical neck dissection, which revealed additional metastases in 2 of 21 lymph nodes. Subsequently, she underwent a diagnostic I-123 scan, which revealed trace uptake in the thyroid bed and faint uptake in the contralateral/left neck despite the negative preoperative nodal ultrasound. This scan was followed by definitive RAI ablation using 152.5 mCi of I-131. She is presently without evidence of disease 1 year after her neck dissection.

## 3. Background

RAI has been used in the management of well-differentiated thyroid cancer since the 1940s. After thyroidectomy, postoperative radioiodine is used to ablate a thyroid remnant, eliminate suspected micrometastases, or eliminate known persistent disease. RAI is also used diagnostically for localization and uptake before ablation therapy. The efficacy of radioiodine depends on patient preparation, tumor-specific characteristics, sites of disease, and dosage. Listed in order of increasing aggressive behavior, the histologic variants of well-differentiated, poorly differentiated, and anaplastic thyroid cancers can be seen as a spectrum of progression. As their aggressive behavior increases, the ability of RAI uptake decreases.

Based on guidelines set by the American Thyroid Association, evidence for RAI effectiveness is only available for patients with age >45 years old with tumor size >4 cm, and patients of any age with gross extrathyroidal extension (T4 disease), or any patient with distant metastasis [2]. On the other hand, current evidence indicates that RAI is not effective in T1a tumors (microcarcinomas, <1 cm). For all patients in between these extremes, evidence for RAI effectiveness is largely inconclusive, conflicting, or lacking [3]. High risk features of thyroid cancer include gross extrathyroidal extension, age >45 years, size >4 cm, distant metastasis.  Table 1 lists the indications for RAI and Table 2 lists the present staging system for thyroid cancer.

The use of radioactive iodine-131 (I-131) ablation after thyroidectomy has shown reduced recurrence rates and prolonged survival in all patients with papillary thyroid cancer who are high risk for recurrent disease and has been accepted as part of the standard of care for these patients [4, 5]. However, for low-risk patients, the long-term prognosis after surgery alone is so favorable that I-131 ablation is not usually recommended [5–7].  Table 3 describes the major factors impacting decision making in radioiodine remnant ablation.

Several histological features may place the patient at higher risk of local recurrence or metastases than would have been predicted by the American Joint Commission on Cancer (AJCC) staging system. These include worrisome histologic subtypes (such as tall cell, columnar and insular, as well as poorly differentiated thyroid cancer), the presence of intrathyroidal vascular invasion, or the finding of gross or microscopic multifocal disease. While many of these features have been associated with increased risk, there are inadequate data to determine whether RAI ablation has a benefit based on specific histologic findings, independent of tumor size, lymph node status, and the age of the patient. Therefore, while RAI ablation is not recommended for all patients with these higher risk histologic features, the presence of these features in combination with size of the tumor, lymph node status, and patient age may increase the risk of recurrence or metastatic spread to a degree that is high enough to warrant RAI ablation in selected patients. Tables 4 and 5 depict two sets of criteria commonly used to classify risk of well-differentiated thyroid carcinoma.

## 4. Mechanism of Radioiodine

**Figure 1:**
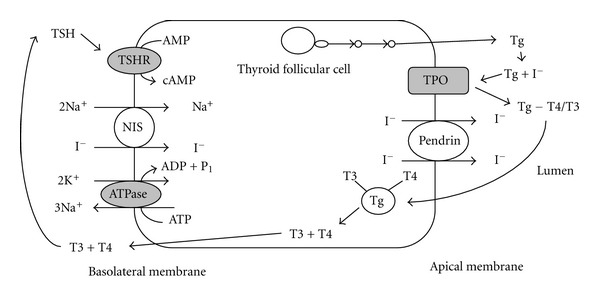
Thyroid follicular cell.

Radioiodine is taken up and concentrated in thyroid follicular cells via a membrane sodium-iodide transporter (Figure 1). Although the sodium-iodide symporter (NIS) is in the thyroid tissue, it is also found in several other tissues that capture iodine such as the stomach, salivary glands, lactating breasts, thymus, nasal mucosa, lacrimal glands, and placenta [8–10]. Differentiated thyroid carcinomas can concentrate iodine, express thyroid stimulating hormone (TSH) receptor, and produce thyroglobulin (Tg), whereas poorly differentiated or undifferentiated carcinomas typically do not. Patients with medullary cancer, lymphoma, and anaplastic cancer are not candidates for RAI because these cancers do not concentrate iodine.

Iodine-131 must be taken up by thyroid tissue to be effective. Thyrotropin is required to stimulate the uptake of radioiodine. The uptake of radioiodine is dependent upon adequate stimulation by TSH, and is suppressed by increased endogenous or exogenous iodide stores. For example, the use of intravenous contrast in CT scans contains a large iodine load which may interfere with RAI scanning and therapy for several months. While several iodine isotopes exist, the most effective form used in adjuvant treatment for papillary thyroid cancer is I-131 [11]. The efficacy of I-131 also depends on the amount of thyroid tissue left behind at surgery.

## 5. Dietary Modifications

Radioiodine uptake is reduced by the presence of excess stable iodide. When planning radioiodine imaging and treatment, the patient should be instructed to avoid all iodine-containing medications and to limit dietary intake of iodine for 1-2 weeks prior to the study. A low-iodine diet is started to maximize uptake and retention of radioiodine by the remnant tumor cells. Per the 2009 American Thyroid Association (ATA) Guidelines, a low-iodine diet for 7–10 days before and for 1-2 days after I-131 administration is suggested to maximize uptake of RAI into thyroid cells, although sufficient evidence is lacking.

The following foods should be avoided for two weeks before I-131 scanning and treatment: iodized salt or sea salt, milk or other dairy products, eggs, seafood (especially shellfish, kelp, or seaweed), any item with added carrageen, agar, algin, or alginates; cured foods; breads made from iodate dough conditioners; foods and medicine containing red food dyes; chocolate; molasses; soy products; restaurant foods and Asian food; pizza; chili. The following foods are allowed: fresh meat, poultry, potatoes or rice, wheat or rye bread, fresh or frozen vegetables, fresh or frozen fruit.

## 6. Optimizing RAI Uptake

Following thyroidectomy, the patient's serum thyroxine (T4) concentration must decline sufficiently to allow the serum TSH concentration to rise above 25–30 mU/L, which is the TSH level necessary to stimulate thyroid tissue for adequate radioiodine uptake by the residual normal follicular cells and tumor cells. The standard approach to raising TSH levels for adequate radioiodine uptake is thyroid hormone withdrawal. Traditionally, levothyroxine is discontinued for approximately 2-3 weeks to achieve thyroid hormone withdrawal, followed by rechecking TSH and proceeding with RAI scanning or ablation.

Alternatively, the symptoms of hypothyroidism that result from thyroid hormone withdrawal can be minimized by giving tri-iodothyronine (short-acting T3 hormone) postoperatively for 3–6 weeks and withdrawing for 1-2 weeks prior to ablation therapy. The serum TSH concentration should rise to 25–30 mU/L within 1-2 weeks after cessation of T3. Another method to minimize hypothyroid symptoms involves the reduction of the dose of oral T4 by 50% for approximately one month rather than stopping it entirely. The goal TSH concentration can be achieved by five weeks, with milder hypothyroid symptoms compared to patients undergoing standard T3 withdrawal.

Another method of preparing patients is to administer recombinant human TSH for RAI ablation.

Recombinant human TSH alpha (trade name Thyrogen; thyrotropin alfa for injection) is a synthetic drug that was developed to provide TSH stimulation with subsequent radioiodine uptake without withdrawal of thyroid hormone and the associated symptoms and morbidity. It also has no adverse cardiovascular effects. Stimulation with rhTSH is known to increase I-131 uptake and Tg release from the tumor remnants and/or metastases. Thyrogen is indicated for use as an adjunctive diagnostic tool for serum Tg testing with or without radioiodine imaging in the follow-up of patients with well-differentiated thyroid cancer who have undergone a near total or total thyroidectomy.

Tg production and RAI uptake, although both associated with degree of differentiation, are unrelated events, that may or may not be present or absent simultaneously. While thyroxine withdrawal or recombinant TSH stimulation is used to increase effectiveness of RAI treatment, stimulated Tg testing is largely used for improvement of the sensitivity of Tg follow up. TSH stimulation by either thyroid hormone withdrawal or administration of recombinant human TSH (rhTSH) is used to enhance the sensitivity of serum Tg in the detection of persistent or recurrent thyroid cancer. For each scan, the patient's serum TSH concentration must be high enough to maximize the uptake of I-131 by any residual thyroid tissue. Since Tg production and release from the cells are significantly influenced by the degree of TSH stimulation, serum Tg measurements are likely to be more sensitive for detection of recurrent disease following TSH stimulation using endogenous TSH or recombinant human TSH, than when measured during TSH suppression.

## 7. Preablation Imaging

Obtaining diagnostic radioiodine scans prior to I-131 radiotherapy is controversial and not routinely recommended by the ATA [2]. There is an increasing trend to avoid pre-therapy RAI scans altogether because of its low impact on the decision to ablate, as well as the concern for stunning of normal thyroid remnants and distant metastases from thyroid cancer. However, the ATA's final recommendation is that pretherapy scans and/or measurement of thyroid bed uptake may be useful when the extent of the thyroid remnant cannot be accurately ascertained from the surgical report or neck ultrasonography, or when the results would alter either the decision to treat or the activity of RAI that is given.

The primary purpose of obtaining a diagnostic radioiodine scan with a low dose of I-131 or I-123 would be to determine the amount of uptake in the postsurgical thyroid bed to assess for remnant disease and to detect distant iodine-avid metastases, which would alter the ablative dose of I-131 administered [12]. Preablation scanning is important for several reasons. First, a negative scan after total thyroidectomy coupled with the absence of measurable serum Tg can identify patients who do not need RAI therapy. Second, the scan allows thorough staging and may reveal metastatic disease that requires a higher therapeutic amount of radioiodine. Finally, some areas of iodine-concentrating metastatic disease may require special preparation before the RAI is administered [13].

Detection of residual tissue after thyroidectomy for papillary or follicular thyroid carcinoma may be performed using diagnostic imaging with either I-123 or I-131 [13–16]. Iodine-123 is more expensive, while I-131 is associated with “stunning,” defined as the reduction in uptake of the therapeutic dose of I-131 caused by some form of cell damage from the diagnostic dosage of I-131 in excess of 74 MBq; this dosage has interfered in some way with the trapping or retention of the therapeutic dosage, or the targeted mass has been reduced in volume and hence in uptake [13, 17]. Several studies have found no evidence of stunning on ablation rate with I-131 diagnostic activities up to 185 MBq, while others have noted a stunning effect with activities of 111–185 MBq [13, 18, 19]. A prospective study examining the outcome of ablative I-131 therapy after diagnostic studies was performed to determine if diagnostic dosages of either I-123 or I-131 reduced the efficacy of I-131 given for remnant ablation [13]. Diagnostic dosages of 14.8 MBq of I-123 or of 74 MBq of I-131 were used. The study concluded that 74 MBq of I-131 is an appropriate dosage that yields the same degree of thyroid remnant ablation as 14.8 MBq of I-123, which was confirmed by follow-up scan at 6–8 months and simultaneous undetectable level of serum Tg [13].

Nearly all patients with distant metastases have high serum Tg concentrations and two-thirds of these patients have I-131 uptake in the metastases [6]. Despite this, several series have demonstrated that patients with detectable serum Tg levels may have a negative I-131 WBS [20, 21]. Some studies have shown that the administration of high I-131 activity (100 mCi or more) increases the sensitivity of a WBS performed a few days later and allows the detection of neoplastic foci not seen with diagnostic doses of I-131 [22, 23]. A retrospective study evaluated patients with positive Tg with a negative diagnostic WBS and compared patients untreated or treated with high-131 activities [20]. The authors found that in patients with detectable serum Tg with negative diagnostic WBS, high dose of I-131 therapy may have a therapeutic utility in patients with lung metastases and, to a lesser extent, in those with lymph node metastases. Frequently the Tg values normalized in untreated patients, leading the authors to conclude that treatment with I-131 should be considered according to the results of the first posttherapy scan. They recommended that I-131 treatment be continued up to total remission in patients with posttherapy scan positive in the lung; surgical treatment for patients with node metastases, and no treatment be administered for patients with thyroid bed uptake or no uptake.

In some patients, there may be an elevation of TSH-stimulated Tg, in conjunction with a negative diagnostic I-131 WBS. ^18^F-fluorodeoxyglucose-positron emission tomography (FDG-PET) can be useful for the detection of thyroid cancer recurrence or metastases in these patients [24]. However, whether FDG-PET scanning for the detection of Tg-positive and RAI scan-negative metastases should be performed under TSH stimulation or suppression remains controversial. A recent meta-analysis by Ma and colleagues sought to address this issue [25]. The investigators studied seven prospective controlled clinical trials including 168 patients. PET scans under TSH stimulation versus thyroid hormone suppression showed statistically significant differences in the number of patients with PET true-positive lesions (odds ratio (OR) 2.45, 95% confidence interval (CI) 1.23–4.90) and in the number of the PET-detected lesions (OR 4.92, 95% CI 2.70–8.95) and tumor-to-background ratios. PET scans taken under TSH stimulation altered clinical management in 12/130 (9%) patients in five paired studies (OR 2.40, 95% CI 1.11–5.22). The authors concluded that TSH stimulation significantly improves the sensitivity of FDG-PET in the detection of thyroid cancer recurrence and metastases in patients with elevated Tg levels and iodine-negative disease, and recommended that TSH stimulation should be used for patients undergoing PET scanning in these circumstances.

## 8. Postoperative RAI Treatment

RAI ablation refers to the total destruction of residual macroscopically normal thyroid tissue after complete gross surgical resection of cancer. Although ablation of the remaining lobe with radioactive iodine has been used as an alternative to completion thyroidectomy, the ATA does not recommend routine radioactive iodine ablation in lieu of completion thyroidectomy. RAI in the form of I-131 is given postoperatively for three reasons [5–7, 26]. First, it destroys any remaining normal thyroid tissue, thereby increasing the sensitivity of subsequent I-131 total-body scanning and the specificity of serum thyroglobulin measurements for the detection of persistent or recurrent disease. Second, iodine-131 therapy can destroy occult microscopic carcinoma, subsequently decreasing the long-term risk of recurrent disease. Third, the use of a large amount of I-131 for therapy permits postablative I-131 total-body scanning, a sensitive test for detecting persistent carcinoma.

Ablative therapy is given 4–6 weeks after thyroidectomy. Based upon concerns about dose-dependent complications after RAI therapy, the ATA guidelines recommend using the minimal activity (30 to 100 mCi) necessary to achieve successful ablation [2]. Iodine-131 is administered using a fixed-activity (fixed-dose) regimen, typically between 29 and 150 mCi (1073 and 5550 MBq). 30 mCi dose of I-131 can be given on an outpatient basis and is successful in ablating residual thyroid cells in 80% of patients. Treatment with higher administered activities of I-131 is used for patients with residual postoperative disease in the thyroid bed or in local regional lymph nodes. Dosing approaches also vary for patients with pulmonary metastases, skeletal disease, renal failure, or hemodialysis.

Successful remnant ablation is usually defined as an absence of visible RAI uptake on subsequent diagnostic RAI scan or an undetectable stimulated serum Tg. Tumor uptake of the treatment dose of I-131 is confirmed by performing a WBS 2 to 10 days after the therapeutic dose. In about 20 percent of these posttreatment scans, foci of uptake that were not seen on the corresponding low-dose I-131 diagnostic scan are seen [27, 28]. However, in only 10 percent of cases does the posttreatment scan reveal new sites of uptake that significantly alter the patient's prognosis and were not known to exist by other means, such as radiography or surgery. Similarly, in a comparison of diagnostic I-123 scans with posttreatment scans, additional areas of uptake were found in 6 percent of patients scanned for the first time, 18 percent of patients scanned for the second time, and 44 percent of patients with high serum Tg concentrations and negative diagnostic scans; however, treatment was altered in only a few patients [29]. The clinical utility of these scans is lowest in older patients receiving their first I-131 treatment; however, the information obtained on the post-therapy scan can guide followup recommendations and should be done routinely. If residual thyroid tissue remains following the first ablative therapy, a second dose of 30 mCi of I-131 may be given 6–12 months later.

## 9. Low-Dose versus High-Dose RAI

There is uncertainty over radioiodine dosage required for effective ablation. The administered activity varies widely between centers from as low as 925 MBq (25 mCi) to as high as 7400 MBq (200 mCi) regardless of whether chosen empirically or based on dosimetry-guided techniques [3, 30]. The United Kingdom 2007 guidelines recommend the use of high-dose radioiodine, while the ATA 2009 guidelines and the European 2006 consensus report suggest that clinicians can choose between the low dose and the high dose [2, 31, 32]. The National Comprehensive Cancer Network (NCCN) guidelines recommend using 30 to 100 mCi radioiodine in cases of papillary, follicular, or Hurthle cell carcinoma ≥1 cm in diameter, with nodal or distant metastases or with aggressive histology when there is suspected or proven thyroid bed uptake in total body radioiodine scan after thyroidectomy [33].

A randomized study on thyroid ablation following thyroidectomy for differentiated thyroid cancer evaluated the safety and efficacy of two commonly used radioiodine ablative activities (1100 MBq, 30 mCi; 3700 MBq, 100 mCi) [3]. There was no conclusive evidence that higher iodine dose (3700 MBq) is more often associated with successful thyroid remnant ablation than the smaller dose (1100 MBq) when administered following thyroidectomy. The smaller administered radioiodine activity was generally better tolerated. In the corresponding meta-analysis, the success rate of thyroid remnant ablation as evaluated by radioiodine scan tended to be higher with the 3700 MBq activity than with the 1100 MBq activity. Similarly, other randomized trials have compared 1100 MBq activity with 1800 activity, or 1800 MBq activity with 3700 MBq activity and suggested a high activity is more efficacious; however, the pooled risk ratios are not statistically significant [3, 34–36].

The relative benefits and harms of using high (3700 MBq) as compared to low (1100 MBq) radioiodine activity remain inadequately studied [3]. A randomized noninferiority trial was performed to determine whether low-dose radioiodine (1100 MBq) could be used instead of high-dose radioiodine (3700 MBq) and whether patients could receive thyrotropin alfa before ablation instead of thyroid hormone withdrawal [12]. The study concluded that low-dose radioiodine plus thyrotropin alfa is an effective and convenient treatment with reduced radiation exposure. Additionally, the use of reduced dose of radioiodine has important advantages including reduced financial costs, reduced exposure to radioactive iodine in the environment, less time in hospital isolation, and fewer side effects.

## 10. Postablation Scanning

Typically a repeat radioiodine diagnostic scan with I-123 or I-131 is recommended 6 to 12 months after surgery in moderate-to-high-risk patients and in lower risk patients who have detectable Tg that are not declining [33]. If significant uptake is seen within the thyroid bed (>1%), one more treatment with 100 to 150 mCi (3.7 to 5.550 GBq) of I-131 may be given to complete the ablation; generally, if there is only minor thyroid bed uptake (<1%) in the absence of clear evidence of residual cancer by other imaging techniques such as ultrasound, then treatment is not repeated. If there is uptake outside of the thyroid bed, then doses of I-131 are given that are appropriate to the site of uptake.

Success of ablation is a prognostic factor for disease-free interval and survival in differentiated thyroid cancer patients [37]. Total ablation is verified by performing I-131 total body scanning 6–12 months later with 2 mCi. Unsuccessful ablation carries a considerably higher risk of recurrence; patients may never, or only after a number of additional therapeutic dosages of I-131, become free of detectable disease. These patients should be followed much more intensively than patients with successful ablation with one dosage, because they may never become disease-free and have a high risk of recurrence.

The use of recombinant human TSH (rhTSH) to stimulate thyroid tissue instead of thyroid hormone withdrawal in preparation for post-treatment scanning has been studied [38]. In one study, 127 patients with thyroid cancer underwent whole body radioiodine scanning by two techniques: first after receiving two doses of rhTSH while T4 was continued and second after the withdrawal of T4. The sensitivity of scanning for the detection of uptake was higher after withdrawal than after rhTSH administration. In contrast, a retrospective analysis of 289 patients at a single institution reported that whole body scan (WBS) results and stimulated serum Tg concentrations were similar after thyroxine withdrawal or administration of two doses of rhTSH [39]. Clearance of radioiodine is decreased during the hypothyroid withdrawal phase when compared with the euthyroid rhTSH phase, which may explain some of the difference in sensitivity between the two techniques, as well as the twofold increase in whole body retention of radioiodine 48 hours after a radioiodine dose [40]. This difference in clearance can result in scans with lower counts after rhTSH, but also reduces whole body radiation exposures.

Thyroglobulin is a glycoprotein that is produced only by normal or neoplastic follicular cells. Serum Tg levels are used to detect the presence or absence of residual, recurrent or metastatic disease in patients with differentiated thyroid cancer, though an elevated Tg measurement is less reliable than FNAB-proven evidence of recurrent disease [41, 42]. Serum Tg levels are usually undetectable in patients without residual disease during the postsurgical followup of differentiated thyroid carcinoma. In 10–15% of patients there is a discrepancy; serum Tg levels may be detectable in patients with no evidence of residual or metastatic thyroid tissue, including a negative diagnostic WBS [20]. Additionally, serum Tg levels may be falsely negative in patients found to have elevated anti-thyroglobulin antibodies. Serum Tg appears to be influenced by the anatomic location, specific histology of the lesion, and volume of disease in patients with metastatic thyroid cancer.

A common approach prior to incorporating stimulated Tg testing into diagnostic algorithms was to continue scanning to obtain two successive negative I-131 scans; this predicted a 97 percent 10-year relapse-free survival compared with 91 percent after only one negative scan [43]. Since the development of stimulated Tg testing, either by hormone withdrawal or thyrotropin alpha, a single negative scan/Tg combination may be sufficient in Stage III or IV patients, and lower risk patients with negative Tg levels may not require follow-up scanning at all. A diagnostic total body scan at 6 to 12 months is actually no longer routinely performed in patients with a normal total-body scan after therapy [44–46]. RAI treatment is not needed for patients with Tg levels <1 ng/mL, negative radioiodine imaging, and negative anti-Tg antibodies.

## 11. ContraIndications

Absolute contraindications to RAI therapy include pregnancy and breastfeeding. Radioiodine can destroy fetal thyroid tissue and result in cretinism [6, 47]. Pregnant women should never be treated with I-131. Furthermore, pregnancy should also be delayed for at least 6 months after receiving RAI therapy. Estrogenized breast tissue also has increased sodium-iodide symporter activity; the lactating breast concentrates a substantial amount of iodide. Breast-feeding should be discontinued to prevent I-131 in the milk from reaching the infant, as well as to limit the radiation of breast tissue. Breastfeeding must be stopped at least 6–8 weeks prior to RAI therapy to reduce uptake by breast tissue.

## 12. Complications

RAI can be associated with significant sequelae. Short-term complications occur in 10–30% of patients and include radiation thyroiditis, painless neck edema, sialadenitis and tumor hemorrhage or edema [6]. These acute side effects are usually mild and resolve rapidly. After I-131 treatment, men may also have a transient reduction in spermatogenesis, and women may have transient ovarian failure. The increase in frequency of miscarriage during the year preceding conception has led to the recommendation of postponing conception until one year after treatment with I-131.

The association of RAI therapy with the development of a second neoplasia in patients with DTC has been controversial. The National Cancer Institute's (NCI) Surveillance, Epidemiology and End Results (SEER) Program is a population-based registry of 26% of cancers diagnosed in the United States. Quality control is an integral part of SEER, which includes information related to RAI therapy. Using the SEER database, the patterns of RAI use and risk of secondary primary malignancies (SPMs) were assessed in patients with low-risk (T1N0) well-differentiated thyroid cancer [48]. There was an increased risk of SPM in these patients, which combined with a lack of data demonstrating an improved survival outcome with adjuvant RAI led to the recommendation of rationing the use of RAI in this patient population.

The three most common nonsynchronous second primary malignancies (NSPMs) are primary breast, colon and lung cancers in patients with DTC, whether or not radioiodine therapy is given. Some studies have shown an increased risk for a second malignancy related to iodine therapy [49–51]. Other studies have suggested that patients with DTC show an increased incidence of neoplasms that are unrelated to I-131 therapy [52, 53].

High cumulative doses of RAI may be associated with a statistically significant increase in SPMs. The absolute risk for radioiodine-induced cancers has not been well established, but the risk of any SPM after initial diagnosis of thyroid cancer is increased approximately 30% over that of the general population and the risk appears to increase with increasing cumulative administered activity [50, 51]. Differentiated thyroid cancer survivors are at increased risk of NSPM, although the cause remains unclear. In one study, the 20-year cumulative risk of NSPM was significantly higher in patients that received radioiodine compared to those that did not (13.5% versus 3.1%, *P* = 0.015) [54]. Although I-131 is preferentially taken up by normal and malignant thyroid follicular cells, it is also taken up and accumulated into the stomach, salivary glands, colon, and bladder; salivary glands and estrogenized breast tissue contain sodium iodide transporters and radioiodine undergoes gastrointestinal and urinary excretion. The risk of salivary gland, breast, bladder and gastrointestinal cancers can theoretically occur more frequently in thyroid cancer patients treated with radioiodine. This occurs between 2 and 10 years after therapy with a prevalence of about 0.5%. Cumulative RAI activity was found to be the only independent risk factor for NSPM in radiation-naive DTC survivors [49, 50].

A study evaluating the frequency of another primary malignancy and its temporal relation to I-131 found that patients with DTC could have increased incidence of a second neoplasia which is not related to I-131 therapy, suggesting a common etiology and or genetic mechanism rather than a causal relationship between the two tumors [8]. In one study, the only parameter that showed statistically significant differences was age at thyroidectomy; this suggests that at the time of surgery, older patients could be at increased risk for developing a second malignancy [52, 53].

## 13. Return to Clinical Scenario

“Are the present guidelines on the use of RAI adequate or should the threshold for RAI use be decreased? Should RAI have been utilized sooner in our case, after the patient's completion thyroidectomy?”

The effectiveness of RAI ablation decreasing recurrence and possibly mortality in low-risk patients with well-differentiated thyroid carcinoma after thyroidectomy has not been clearly determined [46, 55–57]. The ATA guidelines suggest that routine follow-up diagnostic WBS one year after RAI ablation is not even required in low-risk patients [2]. Given the risks associated with RAI, efforts have been made to reduce unnecessary administration in patients not expected to benefit from treatment. In these low-risk patients, a combination of neck ultrasound and rhTSH-stimulated serum Tg may be effective and adequate for detecting persistent or recurrent disease.

In a randomized trial comparing two thyrotropin-stimulation methods and two radioiodine-131 doses, complete ablation was achieved in 631 of 684 patients. The patients were of the following stages: pT1 (tumor diameter ≤ 1 cm) and N1 or Nx, pT1 (tumor diameter > 1 to 2 cm) and any N stage, or pT2N0 [465]. The authors concluded that the use of recombinant human thyrotropin and a low dose of I-131 for postoperative RAI ablation represented an effective and attractive option for the management of low-risk thyroid cancer that reduced the amount of whole-body irradiation.

Defining optimal therapy for patients with low-risk thyroid cancer is challenging because of the slow growth of such tumors and the high success rate of initial surgery. A randomized trial with low-risk patients to receive either low-dose radioiodine or no radioiodine would be helpful to monitor for recurrence [58]. Any therapeutic radioiodine intervention should at least be nontoxic. Until these studies are done, close surveillance including serum testing as well as cervical ultrasound will remain crucial in the postoperative care of all patients, even those considered low-risk, and in facilities that do not offer RAI. In the meantime, the decision for RAI ablation must be individualized, based on the risk profile of the patient, as well as patient and physician preference, while balancing the risk and benefits of RAI therapy and maintaining a good quality of life.

## Figures and Tables

**Table 1:** Indications for RAI (adapted from NCCN 2012 guidelines).

Recommended	
(i) All patients with gross extrathyroidal extension, primary tumor size > 4 cm, distant metastases	Papillary Follicular Hurthle
(ii) For select patients without gross residual disease when the combination of clinical factors predicts an intermediate to-high-risk for recurrence or disease-specific mortality (e.g., primary tumors ranging from 1–4 cm confined to the thyroid, high-risk histologies, vascular invasion, or cervical lymph node metastases)	Papillary

Not recommended	

(i) Not routinely recommended for patients with either unifocal or multifocal papillary microcarcinomas (<1 cm) confined to the thyroid	Papillary
(ii) Not required: minimally invasive follicular thyroid carcinoma or Hurthle cell carcinoma confined to the thyroid when the primary tumor is small and demonstrates only invasion of the tumor capsule without vascular invasion	Follicular Hurthle

**Table 2:** AJCC staging, 7th ed., for well-differentiated thyroid cancer based on TNM descriptors [2].

T1	Tumor diameter ≤ 2 cm, limited to the thyroid	
T2	Primary tumor diameter > 2 cm to 4 cm, limited to the thyroid	
T3	Primary tumor diameter > 4 cm, limited to the thyroid or with minimal extrathyroidal extension	
T4a	Tumor of any size extends beyond thyroid capsule to invade subcutaneous soft tissues, larynx, trachea, esophagus, or recurrent laryngeal nerve	
T4b	Tumor invades prevertebral fascia or encases carotid artery or mediastinal vessels	
TX	Primary tumor size unknown, but without extrathyroidal invasion	

N0	No regional lymph node metastasis	
N1a	Metastasis to level VI (pretracheal, paratracheal, and prelaryngeal/Delphian lymph nodes)	
N1b	Metastasis to unilateral, bilateral, contralateral, cervical, or superior mediastinal lymph nodes	
NX	Nodes not assessed at surgery	

M0	No distant metastases	
M1	Distant metastases	
MX	Distant metastases not assessed	

	Patient age < 45 years	Patient age ≥ 45 years

Stage I	Any T, any N, M0	T1, N0, M0
Stage II	Any T, any N, M1	T2, N0, M0
Stage III		T3, N0, M0
		T1, N1a, M0
		T2, N1a, M0
		T3, N1a, M0
Stage IVA		T4a, N0, M0
		T4a, N1a, M0
		T1, N1b, M0
		T2, N1b, M0
		T3, N1b, M0
		T4a, N1b, M0
Stage IVB		T4b, Any N, M0
Stage IVC		Any T, Any N, M1

**Table 3:** Major factors impacting decision making in radioiodine remnant ablation, 2009 ATA guideline [2].

Factors	Description	Expected Benefit				
		Decreased risk of death	Decreased risk of recurrence	May facilitate initial staging and followup	RAI ablation usually recommended	Strength of evidence
T1	≤1 cm, intrathyroidal or microscopic multifocal	No	No	Yes	No	E
	1-2 cm, intrathyroidal	No	Conflicting data	Yes	Selective use	I
T2	>2–4 cm, intrathyroidal	No	Conflicting data	Yes	Selective use	C
T3	>4 cm,					
	<45 years	No	Conflicting data	Yes	Yes	B
	≥45 years	Yes	Yes	Yes	Yes	B
	Any size, any age, minimal extrathyroidal extension	No	Inadequate data	Yes	Selective use	I
T4	Any size with gross extrathyroidal extension	Yes	Yes	Yes	Yes	B
NX, N0	No metastatic nodes documented	No	No	Yes	No	I
N1	<45 years	No	Conflicting data	Yes	Selective use	C
	>45 years	Conflicting data	Conflicting data	Yes	Selective use	C
M1	Distant metastases present	Yes	Yes	Yes	Yes	A

**Table 4:** ATA risk of recurrence classification after initial surgery [2].

Low risk
(i) No local or distant mets
(ii) All macroscopic tumor has been resected
(iii) There is no tumor invasion of locoregional tissues or structures
(iv) Tumor does not have aggressive histology (e.g., tall cell, insular, columnar cell carcinoma) or vascular invasion
(v) If I-131 is given, there is no I-131 uptake outside the thyroid bed on the first posttreatment whole-body RAI scan

Intermediate risk

(i) Microscopic invasion of tumor into the perithyroidal soft tissue at initial surgery
(ii) Cervical LN mets or I-131 uptake outside the thyroid bed on the post-treatment whole-body RAI scan done after thyroid
remnant ablation
(iii) Tumor with aggressive history or vascular invasion

High risk

(i) Macroscopic tumor invasion
(ii) Incomplete tumor resection
(iii) Distant mets
(iv) Possibly thyroglobulinemia out of proportion to what is seen on the post-treatment scan

**Table 5:** Criteria for distinguishing low-risk and high-risk well-differentiated carcinoma based on the AGES (age, grade, extrathyroid extent, and size) classification system.

Low	High
Women < 50 years	Women ≥ 50 years
Men < 40 years	Men ≥ 40 years
Well-differentiated tumor	Poorly differentiated tumor (tall cell, columnar cell, or oxyphilic variants)
Tumor < 4 cm in diameter	Tumors ≥ 4 cm in diameter
Tumor confined to thyroid	Local invasion
No distant metastases	Distant metastases

## Acronyms

AGES: Age, grade, extrathyroid extent and size
AJCC: American Joint Commission on Cancer
ATA: American Thyroid Association
CT: Computed tomography
DTC: Differentiated thyroid carcinoma
FAP: Familial adenomatous polyposis
FDG: Fludeoxyglucose
FNA: Fine needle aspiration
I-123: Iodine-123
I-131: Iodine-131
MEN2: Multiple endocrine neoplasia type 2
NCCN: National Comprehensive Cancer Network
NIS: Sodium-iodide symporter
NSPM: Nonsynchronous second primary malignancy
PET: Positron emission tomography
RAI: Radioactive iodine
rhTSH: Recombinant human thyroid-stimulating hormone
T3: Tri-iodothyronine
T4: Thyroxine
Tg: Thyroglobulin
TPO: Thyroid peroxidase
TSH: Thyroid-stimulating hormone
TSHR: Thyroid-stimulating hormone receptor
WBS: Whole-body scan.
